# Risk of Psychiatric Disorders following Irritable Bowel Syndrome: A Nationwide Population-Based Cohort Study

**DOI:** 10.1371/journal.pone.0133283

**Published:** 2015-07-29

**Authors:** Yao-Tung Lee, Li-Yu Hu, Cheng-Che Shen, Min-Wei Huang, Shih-Jen Tsai, Albert C. Yang, Chang-Kuo Hu, Chin-Lin Perng, Yi-Shin Huang, Jeng-Hsiu Hung

**Affiliations:** 1 Department of Psychiatry, Kaohsiung Veterans General Hospital, Kaohsiung, Taiwan; 2 Department of Psychiatry, Taipei Medical University, Shuang-Ho Hospital, New Taipei City, Taiwan; 3 Department of Psychiatry, Chiayi Branch, Taichung Veterans General Hospital, Chiayi, Taiwan; 4 Department of information magagement, National Chung-Cheng University, Chiayi, Taiwan; 5 School of Medicine, National Yang-Ming University, Taipei, Taiwan; 6 Bali Psychiatric Center, Ministry of Health and Welfare, New Taipei, Taiwan; 7 Department of Psychiatry, Taipei Veterans General Hospital, Taipei, Taiwan; 8 Division of Neurosurgery, Department of surgery, Chiayi Branch, Taichung Veterans General Hospital, Chiayi, Taiwan; 9 Division of Gastroenterology, Department of Medicine, Taipei Veterans General Hospital, Taipei, Taiwan; 10 Department of Obstetrics and Gynecology, Taipei Tzu Chi Hospital, Buddhist Tzu Chi Medical Foundation, Taipei, Taiwan; 11 School of Medicine, Tzu Chi University, Hualien, Taiwan; Johns Hopkins Bloomberg School of Public Health, UNITED STATES

## Abstract

**Background:**

Irritable bowel syndrome (IBS) is the most common functional gastrointestinal (GI) disorder observed in patients who visit general practitioners for GI-related complaints. A high prevalence of psychiatric comorbidities, particularly anxiety and depressive disorders, has been reported in patients with IBS. However, a clear temporal relationship between IBS and psychiatric disorders has not been well established.

**Objective:**

We explored the relationship between IBS and the subsequent development of psychiatric disorders including schizophrenia, bipolar disorder, depressive disorder, anxiety disorder, and sleep disorder.

**Methods:**

We selected patients who were diagnosed with IBS caused by gastroenteritis, according to the data in the Taiwan National Health Insurance Research Database. A comparison cohort was formed of patients without IBS who were matched according to age and sex. The incidence rate and the hazard ratios (HRs) of subsequent new-onset psychiatric disorders were calculated for both cohorts, based on psychiatrist diagnoses.

**Results:**

The IBS cohort consisted of 4689 patients, and the comparison cohort comprised 18756 matched control patients without IBS. The risks of depressive disorder (HR = 2.71, 95% confidence interval [CI] = 2.30–3.19), anxiety disorder (HR = 2.89, 95% CI = 2.42–3.46), sleep disorder (HR = 2.47, 95% CI = 2.02–3.02), and bipolar disorder (HR = 2.44, 95% CI = 1.34–4.46) were higher in the IBS cohort than in the comparison cohort. In addition, the incidence of newly diagnosed depressive disorder, anxiety disorder, and sleep disorder remained significantly increased in all of the stratified follow-up durations (0–1, 1–5, ≥5 y).

**Conclusions:**

IBS may increase the risk of subsequent depressive disorder, anxiety disorder, sleep disorder, and bipolar disorder. The risk ratios are highest for these disorders within 1 year of IBS diagnosis, but the risk remains statistically significant for more than 5 years. Clinicians should pay particular attention to psychiatric comorbidities in IBS patients.

## Introduction

Irritable bowel syndrome (IBS) is the most common functional gastrointestinal (GI) disorder, accounting for 50% of patients who visit general practitioners for GI-related complaints [1] and is characterized by chronic abdominal pain, bloating, and alterations in bowel habits, which hamper the life quality of afflicted individuals [2]. It is estimated that IBS affects 10–15% of the population across the world with a female predominance [3, 4] and imposes a substantial economic burden in terms of direct health care costs [5].

Numerous studies have evaluated the relationship between IBS and psychiatric status. Increasing evidence shows that compared with healthy subjects, IBS patients exhibit differences in brain imaging studies, which may suggest the biological association of the psychiatric conditions in IBS [6–9]. Therefore, psychiatric comorbidities may be one of the aspects worthy of IBS-related discussions [10, 11]. According to previous studies, nearly 50–60% of IBS patients experience major psychosocial problems [12, 13]. It has been reported that following acute gastroenteritis, prior anxiety and depression might be risk factors for the subsequent development of post-infectious IBS [14, 15]. In addition, high anxiety and depression scores have been reported in a post-infectious IBS population following initial infection [16]. Therefore, exploring the psychological aspects of IBS is crucial for an obtaining clearer holistic understanding of the syndrome and for developing effective treatments.

Although the above mentioned research has provided insight to the association between IBS and comorbid psychiatric disorders, most of these study results are based on psychiatric ratings rather than diagnosis by a psychiatrist, or based on cross-sectional study design and lack a longitudinal perspective. Furthermore, the small sample size of most of the above studies prevents generalization of the findings.

So far, there is a lack of national data with very few longitudinal studies regarding between IBS and risk of psychiatric disorders. Due to this fact, and also on the hypothesis that IBS patients may have a higher risk of developing psychiatric disorders, we have planned a nationwide population-based retrospective cohort study to investigate the possible link between these two illnesses.

## Patients and Methods

### Data source

The National Health Insurance Research Database (NHIRD) in Taiwan, which contains registry files and all medical benefit claims for approximately 25.68 million enrollees, was established in 1998. Numerous researchers worldwide have used the NHIRD for published studies. The NHIRD contains complete information regarding clinical visits, including prescription elements and diagnostic codes based on the A code and the International Classification of Diseases, Ninth Revision, Clinical Modification (ICD-9-CM). The NHIRD is managed and openly released for research purposes by the National Health Research Institutes (NHRI), with confidentiality being maintained according to the directives of the Bureau of the NHI. The Longitudinal Health Insurance Database 2005 (LHID 2005) was used in this study. LHID 2005 contains all original claim data of 1,000,000 beneficiaries which were randomly sampled from the year 2005 Registry for Beneficiaries of the NHIRD, where registration data of everyone who was a beneficiary of the National Health Insurance program during the period of Jan. 1st 2005 to Jan. 1st, 2006 were drawn for random sampling. All the registration and claim data of these 1,000,000 individuals collected by the National Health Insurance program constitute the LHID2005 and we have data of 1,000,000 individuals in LHID 2005 from 1996 to 2009. The NHRI affirms that there are no statistical differences in the distributions of age, sex, or health care costs between the data in the LHID 2005 and that of the NHIRD [17].

### Ethics statement

This study was approved by the Institutional Review Board of Taipei Veterans General Hospital. Written consent was not obtained from the study participants because the data were obtained from the LHID 2005, which contains deidentified secondary data. In addition, the Institutional Review Board issued a formal written waiver for the need for consent.

### Study population

We extracted data from the LHID 2005 for this retrospective cohort study, in which patients newly diagnosed with IBS between January 1, 2000 and December 31, 2008 were selected. The patients with IBS were defined according to the ICD-9-CM code 564.1. To ensure diagnostic validity and patient homogeneity, we included only patients who were diagnosed by gastroenterologists and had at least two consensus IBS diagnoses. We excluded patients who were diagnosed with IBS (ICD-9-CM code: 564.1) between January 1, 1996, and December 31, 1999. In addition, we excluded patients who were diagnosed with psychiatric disorders (A codes: A210-A219; ICD-9-CM codes: 290–319) prior to IBS diagnosis. The index date was defined as the date when an eligible GERD patient was included in our GERD cohort. For each patient with IBS included in the final cohort, four age- and sex-matched patients without IBS and any psychiatric disorder were randomly selected from the LHID 2005 and included in the comparison cohort with the same index date. The random assignment procedures were performed by SAS statistical software and were based on the random numbers which were generated from the uniform distribution. All participants were observed until they were diagnosed with schizophrenia (ICD-9-CM code: 295), depressive disorder (ICD-9-CM codes: 296.2, 296.3, 300.4, and 311), bipolar disorder (ICD-9-CM codes: 296.0, 296.1, 296.4, 296.5, 296.6, 296.7, 296.8, 296.80, and 296.89), anxiety disorder (ICD-9-CM codes: 300.0, 300.2, 300.3, 308.3, and 309.81), or sleep disorder (ICD-9-CM codes: 780.5, 307.4 [excluding 780.51, 780.53, 780.57]); or until death, withdrawal from the NHI system, or December 31, 2009. The primary clinical outcomes were psychiatrist-diagnosed schizophrenia, depressive disorder, bipolar disorder, anxiety disorder, and sleep disorder. Furthermore, common comorbidities, including hypertension, diabetes mellitus, dyslipidemia, congestive heart failure, chronic pulmonary diseases, coronary artery diseases, cerebrovascular diseases, and malignancies, were compared among the participants in the IBS and control cohorts.

### Statistical analysis

The incidence of newly diagnosed schizophrenia, depressive disorder, bipolar disorder, anxiety disorder, or sleep disorder in the IBS and control cases was the primary outcome in this study. We compared the distributions of the demographic characteristics between the two groups by using independent *t*-tests and a chi-squared test. In addition, a Cox proportional hazard regression model was used to calculate the hazard ratios (HRs) of schizophrenia, depressive disorder, bipolar disorder, anxiety disorder, and sleep disorder in the IBS and control cohorts. Furthermore, to investigate potential surveillance bias, subgroups were stratified according to the duration since IBS diagnosis.

The SAS statistical software for Windows, Version 9.3 (SAS Institute, Cary, NC, USA), was used for data extraction, computation, linkage, processing, and sampling. All other statistical analyses were performed using the SPSS statistical software for Windows, Version 20 (IBM, Armonk, NY, USA). *P* < .05 was considered to be statistically significant.

## Results

Our study sample comprised 4689 and 18756 participants in the IBS and control cohorts, respectively. For both cohorts, 56.45% were male and 43.55% were female. Table 1 presents the demographic and clinical variables between the two groups. The median age of the patients was 47.47 years (interquartile range, 34.79–59.09 y) and the median follow-up duration was 5.92 and 5.94 years separately. Hypertension, chronic pulmonary diseases, dyslipidemia, and diabetes mellitus were the four most common comorbidities in both the groups. Baseline differences in comorbidities revealed a higher prevalence of hypertension, diabetes mellitus, dyslipidemia, congestive heart failure, chronic pulmonary diseases, and malignancy among patients in the IBS cohort.

**Table 1:** Characteristics of IBS and control subjects.

	IBS cohort	Control cohort	P value
No.	4689	18756	
Age (years) ^a^	47.47 (34.79–59.09)	47.47 (34.81–59.08)	.986
Distribution of age			
20–39	1758 (37.49)	7032 (37.49)	
40–59	1830 (38.95)	7320 (38.95)	
≥60	1101 (23.48)	4404 (23.48)	
Sex			> .999
Female	2042 (43.55)	8168 (43.55)	
Male	2647 (56.45)	10588 (56.45)	
Comorbidities			
Hypertension	1046 (22.31)	3478 (18.54)	< .001*
Diabetes mellitus	687 (14.65)	1851 (9.87)	< .001*
Dyslipidemia	933(19.90)	2346 (12.51)	< .001*
Coronary artery disease	47 (1.00)	174 (0.93)	.618
Congestive heart failure	137 (2.92)	390 (2.01)	.001*
Cerebrovascular disease	197 (4.20)	658 (3.51)	.025
Chronic pulmonary disease	804(17.15)	1926 (10.27)	< .001*
Malignancy	102 (2.18)	264 (1.41)	< .001*
Income			< .001*
Low	1985 (42.33)	8781 (46.82)	
Medium	1834 (39.11)	7022 (37.44)	
High	870 (18.55)	2953 (15.74)	
Degree of urbanization			.015*
Urban	2901 (61.87)	11333 (60.42)	
Suburban	1332 (28.41)	5573 (29.71)	
Rural	347 (7.40)	1587 (8.46)	
Follow-up, years ^a^	5.92	5.94	.684
Newly diagnosed psychiatric disorders, N (%)			
Schizophrenia	13 (0.28)	29 (0.15)	.083
Bipolar disorder	17 (0.36)	28 (0.15)	.008*
Depressive disorder	239 (5.10)	363 (1.94)	< .001*
Anxiety disorder	205 (4.37)	290 (1.55)	< .001*
Sleep disorder	153 (3.26)	258 (1.38)	< .001*

^a^ Median (interquartile range)

* Statistical significance

During the follow-up period, in the IBS group, the most common subsequent psychiatric disorders were depressive disorder (239 patients), anxiety disorder (205 patients), and sleep disorder (153 patients). The incidence rate for depressive disorder (4.70 vs. 1.74 per 1,000 person-years, respectively), anxiety disorder (4.00 vs. 1.39 per 1,000 person-years, respectively), sleep disorder (2.94 vs. 1.19 per 1,000 person-years, respectively), and bipolar disorder (0.32 vs. 0.13 per 1,000 person-years, respectively) were all significantly higher for the IBS cohort than for the comparison cohort (depressive disorder: risk ratio [RR] = 2.70, 95% confidence interval [CI]  = 2.28–3.19; anxiety disorder: RR = 2.88, 95% CI = 2.40–3.46; sleep disorder: RR = 2.01, 95% CI = 1.73–2.34; bipolar disorder: RR = 2.44, 95% CI = 1.25–4.61) (Table 2).

**Table 2:** The incidence rates of cases of newly diagnosed schizophrenia, bipolar, depressive, anxiety and sleep disorders between patients in the IBS and control cohorts, stratified by the follow-up duration.

	IBS cohort		Control cohort		
Follow-up duration (year)	Incidence rate of Schizophrenia	Per 1000 person-years	Incidence rate of Schizophrenia	Per 1000 person-years	Risk ratio (95% CI)
Overall	13	0.25	29	0.14	1.80 (0.86–3.57)
0–1	1	0.21	3	0.16	1.33 (0.03–16.62)
1–5	9	0.45	15	0.19	2.41 (0.93–5.88)
≥5	3	0.11	11	0.10	1.09 (0.20–4.14)
	IBS cohort		Control cohort		
Follow-up duration (year)	Incidence rate of Bipolar disorder	Per 1 000 person-years	Incidence rate of Bipolar disorder	Per 1 000 person-years	Risk ratio (95% CI)
Overall	17	0.32	28	0.13	2.44 (1.25–4.61)*
0–1	0	0.00	2	0.11	0.00 (0.00–21.32)
1–5	9	0.45	17	0.21	2.13 (0.84–5.04)
≥5	8	0.29	9	0.08	3.57 (1.20–10.43)*
	IBS cohort		Control cohort		
Follow-up duration (year)	Incidence rate of Depressive disorder	Per 1 000 person-years	Incidence rate of Depressive disorder	Per 1 000 person-years	Risk ratio (95% CI)
Overall	239	4.70	363	1.74	2.70 (2.28–3.19)*
0–1	69	14.86	40	2.13	6.96 (4.65–10.55)*
1–5	119	6.12	197	2.47	2.47 (1.95–3.12)*
≥ 5	51	1.90	126	1.14	1.66 (1.18–2.32)*
	IBS cohort		Control cohort		
Follow-up duration (year)	Incidence rate of anxiety disorder	Per 1000 person-years	Incidence rate of anxiety disorder	Per 1 000 person-years	Risk ratio (95% CI)
Overall	205	4.00	290	1.39	2.88 (2.40–3.46)*
0–1	52	11.18	31	1.65	6.76 (4.25–10.91)*
1–5	99	5.06	147	1.84	2.75 (1.11–3.57)*
≥ 5	54	2.00	112	1.01	1.97 (1.40–2.15)*
	IBS cohort		Control cohort		
Follow-up duration (year)	Incidence rate of sleep disorder	Per 1000 person-years	Incidence rate of sleep disorder	Per 1 000 person-years	Risk ratio (95% CI)
Overall	153	2.94	258	1.19	2.01 (1.73–2.34)*
0–1	25	5.35	33	1.34	4.01 (2.21–7.28)*
1–5	73	3.70	129	1.62	2.29 (1.69–3.07)*
≥ 5	55	1.98	96	0.87	2.28 (1.60–3.22)*

CI confidence interval;

* Statistical significance

The Cox proportional hazard regression analysis was conducted to calculate the HR of the newly diagnosed psychiatric disorders for patients in the IBS and control cohorts (Table 3). The results indicated that compared with the patients in the control cohort, those in the IBS cohort exhibited a significantly higher risk of a subsequent depressive disorder (HR = 2.71, 95% CI = 2.30–3.19), bipolar disorder (HR = 2.44, 95% CI = 1.34–4.46), anxiety disorder (HR = 2.89, 95% CI = 2.42–3.46), and sleep disorder (HR = 2.47, 95% CI = 2.02–3.02).

**Table 3:** Hazard ratios of time until diagnosis of psychiatric disorders between patients in the IBS and control cohorts during a 10-year follow-up period.

	HR	95% CI	P value
Schizophrenia	1.80	0.94–3.46	.078
Bipolar disorder	2.44	1.34–4.46	.004*
Depressive disorder	2.71	2.30–3.19	< .001*
Anxiety disorder	2.89	2.42–3.46	< .001*
Sleep disorder	2.47	2.02–3.02	< .001*

HR hazard ratio; CI confidence interval;

* Statistical significance

Furthermore, a subanalysis based on the duration of follow-up revealed that the patients in the IBS cohort had the highest risk ratio for developing depressive disorder, anxiety disorder, and sleep disorder within 1 year of IBS diagnosis. Although the risk of subsequent psychiatric disorders decreases with time, it remains statistically significant for more than 5 years after diagnosis. However, the risk ratio for subsequent bipolar disorder continues to increase with time, but the ratio becomes statistically significant only when the follow-up duration is longer than 5 years. Regarding schizophrenia, the risk ratio was statistically nonsignificant at all times during and after the follow-up duration. Table 2 lists the results of the subanalysis.

## Discussion

Based on our research, this is the first population-based cohort study to evaluate the subsequent psychiatric status of adult patients in IBS and control cohorts. In this study, we included 4689 and 18756 participants in the IBS and control cohorts, respectively, for comparing the median follow-up time of 5.92 and 5.94 years, respectively. The major finding of our study is that the incidences of subsequent depressive disorder, sleep disorder, anxiety disorder, and bipolar disorder were significantly higher in the IBS cohort than in the control cohort. However, the incidence of schizophrenia was statistically nonsignificant in either cohort.

Numerous studies have reported a high prevalence of psychiatric disorders, particularly anxiety and depressive disorders, in patients with IBS [18]. However, there are certain methodological limitations, including a relatively small sample size, lack of demographically matched control group, cross-sectional but not longitudinal study design, and the use of rating scales but not diagnostic evaluation by psychiatrists. Until now, no study has investigated the association between IBS and subsequent psychiatric disorders. Thus, our study was designed to exclude patients with previous psychiatric diagnosis in IBS and control cohorts, and we longitudinally followed up patients to determine the prevalence of subsequent psychiatric disorders. In addition, only gastroenterologist-diagnosed patients with IBS were included in our cohort, to increase the validity of the diagnosis. Similarly, we included only psychiatrist-diagnosed psychiatric disorders in our cohort results.

Several studies have investigated the depression and anxiety statuses of IBS patients, but the results are conflicting. Some studies have reported that IBS was associated with high levels of depression [19–22] or anxiety or both [19–23], whereas others have reported no such association [24, 25]. A study from National Institute of Mental Health focused on the psychiatric comorbidity prevalence of those with or without GI symptoms, including IBS and medically unexplained GI symptoms, had suggested that compared with those reported no GI symptoms, subjects who report at least one of the GI symptoms were significantly more likely to experience lifetime episodes of major depression (7.5% vs 2.9%), panic disorder (2.5% vs 0.7%), or agoraphobia (10.0% vs 3.6%) [26]. In addition, a recent systemic review and meta-analysis included 10 case-control studies and concluded that IBS patients had significantly higher anxiety and depression levels than those of controls [18]. However, most of these studies were cross-sectional and heterogeneous in establishing a temporal relationship between IBS and psychiatric status. Being a cohort study with a nationwide population base, our study indicated that IBS may be a risk factor for subsequent depressive and anxiety disorders. There are some possible explanations for this result. First, although still elusive in the etiology of IBS, studies have speculated that the impaired brain-gut pathway may play a role in IBS [10, 11]. This model suggests that abdominal illnesses may contribute to depression and anxiety and that psychological factors in turn influence physiological factors such as stress activity of the gut via the vagus nerve and sympathetic afferents [27]. Patients with IBS were acutely aware of the type of signal that the gut can send to the brain, long before the concept of a dysregulated brain-gut axis emerged as the favored explanation for their travails [28]. This bidirectional communication system provided the basis for incremental and much needed improvement in our understanding of IBS [29]. Second, studies have shown that the activation of the hypothalamic-pituitary-adrenal (HPA) axis which leads to increases in circulating corticosteroids are essential for the metabolic adaptation to stress [30].Therefore, the HPA axis dysfunction in IBS, which has been documented in many studies [31, 32], may play a significant role in stress vulnerability. In addition, based on the fact that cytokines could have large influence on the HPA axis [33], evidence on the increase of cytokines such as interleukin 1 (IL-1), interleukin 6 (IL-6) and tumor necrosis factor alpha (TNF-α) among the patients with depression and anxiety may further support this theory [34–39]. Third, tryptophan is a metabolic precursor for the neurotransmitter serotonin and research has found that patients with IBS may have abnormal tryptophan catabolism, which contributes to the decreased serotonin production [40] and this result may be linked to several psychiatric conditions such as depression and anxiety [41]. Finally, although brain-derived neurotrophic factor (BDNF) is an important neurotrophin involved in neurogenesis and synaptic plasticity in the brain, and has been implicated in the pathophysiology of several psychiatric disorders [42], BDNF is also abundant in the periphery, and has been shown to be involved in the pathogenesis of IBS [43, 44]. However, the clear role of BDNF on the gut-brain pathway in IBS still remains unknown. Considering the rarity of the cohort studies, further investigations are needed to confirm the underlying mechanisms of the interaction between IBS and depressive or anxiety disorders.

Few studies have assessed the relationship between IBS and bipolar disorder. One community study found no association between bipolar disorder and IBS [45]. Another case study reported that there is an unusual co-occurrence of IBS with bipolar disorder [46]. In addition, a population-based study using the same database as our study but different study design demonstrated that IBS increases the risk of developing subsequent bipolar disorder [47]. In our study, we observed that patients with IBS were at a higher risk of developing bipolar disorder than those without IBS, and the risk was statistically significant after more than 5 years of follow-up. Further study is warranted.

Regarding the relationship between IBS and schizophrenia, evidence has shown that the prevalence of IBS in schizophrenia patients was 19% [48]. However, no study has investigated the prevalence of schizophrenia or the longitudinal relationship between IBS and schizophrenia among IBS patients. In our study, no significantly high risk of subsequent schizophrenia was observed after a diagnosis of IBS.

Moreover, our study indicated that patients with IBS exhibit an increased risk of developing new-onset sleep disorders. At least two factors may link the two aforementioned disorders. First, inflammatory cytokines, such as interleukin-1 and interleukin-6, have been shown to be significant contributors to sleep disturbances. Alterations in these cytokine levels have been demonstrated in certain gastrointestinal diseases including inflammatory bowel disease [49]. Another possible factor is melatonin, which is a hormone produced by the pineal gland and is correlated with the sleep cycle. In addition, high quantities of melatonin are produced by the enterochromaffin cells of the digestive mucosa, which play a crucial role in gastrointestinal physiology, including regulation of gastrointestinal motility, local anti-inflammatory reactions as well as moderation of visceral sensation [50]. Therefore, additional studies should be conducted to investigate the association between IBS and the risk of sleep disorder.

We conducted a subgroup analysis that was stratified according to the duration between the diagnosis of IBS and new-onset psychiatric disorders (Table 2). The results indicated that incident depressive disorder, anxiety disorder, and sleep disorder increased not only within the first year but also the first year or the fifth year after a diagnosis of IBS. Patients with IBS are likely to exhibit a higher frequency of outpatient visits than the general population, leading to an earlier diagnosis of psychiatric disorders which cause surveillance bias [51]. It is also possible that depressive disorder, anxiety disorder, and sleep disorder are the stress reaction to the new diagnosis of IBS. When patients diagnosed with psychiatric disorders within 1 year of IBS diagnosis were excluded, the risk ratio for the newly diagnosed depressive disorder, bipolar disorder, anxiety disorder, and sleep disorder remained high for the IBS cohort, and the ratios were all statistically significant. Thus, this result suggests that the increased risks of depressive disorder, bipolar disorder, anxiety disorder, and sleep disorder in IBS patients were not caused by surveillance bias only.

As shown in Table 2, the risk ratios for anxiety disorder, sleep disorder, and depressive disorder are the highest within 1 year of IBS diagnosis, but they remain statistically significant for more than 5 years after diagnosis. This result may have crucial clinical significance, implying that comorbid psychiatric conditions should be systemically checked and treated in IBS patients. Furthermore, the evaluation should be performed routinely within 1 year of IBS diagnosis and continued thereafter at regular intervals, because the psychological factors are crucial moderators of symptom severity, symptom persistence, treatment-related decisions, and response to treatment [52]. Evidence suggests that psychological intervention may be beneficial in managing gastrointestinal disorders as well as the life quality of the patients, including those in partial remission and with some residual symptoms [53, 54]. Our results suggest that continuous monitoring of the psychiatric condition is essential in IBS patients. Once a psychiatric comorbidity is detected, the patient may receive psychiatric intervention, which may include the use of pharmacotherapy with antidepressants or anxiolytics and psychotherapy. Psychiatric interventions have consistently demonstrated effectiveness in reducing IBS symptoms and improving the functioning of a patient [55].

Our study is one of the few nationwide cohort studies to examine IBS as a risk factor for the development of psychiatric disorders. However, our study has some limitations. First, similar to the etiology of IBS, the pathogenesis of psychiatric disorders is multifactorial. In addition, psychological and environmental factors may play roles in IBS and psychiatric disorders. Several demographic variables were unavailable in our study because our data were obtained from a health care database. Second, according to the current gold standard for diagnosing IBS, the Rome III diagnostic criteria, IBS can be divided into several subgroups [56]. However, in the ICD system, IBS was coded for only one code (564.1), making subgroup analysis impossible. Finally, some of the psychiatric disorders may have a prodromal phase, and the duration of follow-up may be insufficient for detecting late-onset psychiatric disorders. Therefore, future studies should consider these limitations.

In conclusion, our nationwide population-based cohort study indicated an increased risk of depressive disorder, anxiety disorder, sleep disorder, and bipolar disorder among IBS patients. Furthermore, the risk ratios for depressive disorder, anxiety disorder, and sleep disorder are the highest within 1 year of IBS diagnosis, but the risk remains statistically significant for more than 5 years since diagnosis. Future studies and current management practices for IBS patients should consider these findings.
